# COVID-19 Vaccination Strategy in China: A Case Study

**DOI:** 10.3390/epidemiologia2030030

**Published:** 2021-09-03

**Authors:** Marjan Mohamadi, Yuling Lin, Mélissa Vuillet Soit Vulliet, Antoine Flahault, Liudmila Rozanova, Guilhem Fabre

**Affiliations:** 1 Institute of Global Health, University of Geneva, 1211 Geneva, Switzerland; Marjan.Mohamadi@etu.unige.ch (M.M.); Melissa.Vuillet@etu.unige.ch (M.V.S.V.); antoine.flahault@unige.ch (A.F.); liudmila.rozanova@unige.ch (L.R.); 2 Department of Chinese, UFR 2, Université Paul Valéry Montpellier 3, 34199 Montpellier, France; fabreguilhem@orange.fr

**Keywords:** COVID-19, SARS-CoV-2, vaccination strategy, China

## Abstract

The coronavirus disease 2019 (COVID-19) outbreak in China was first reported to the World Health Organization on 31 December 2019, after the first cases were officially identified around 8 December 2019. However, the case of an infected patient of 55 years old can probably be traced back on 17 November. The spreading has been rapid and heterogeneous. Economic, political and social impacts have not been long overdue. This paper, based on English, French and Chinese research in national and international databases, aims to study the COVID-19 situation in China through the management of the outbreak and the Chinese response to vaccination strategy. The coronavirus disease pandemic is under control in China through non-pharmaceutical interventions, and the mass vaccination program has been launched to further prevent the disease and progressed steadily with 483.34 million doses having been administered across the country by 21 May 2021. China is also acting as an important player in the development and production of SARS-CoV-2 vaccines.

## 1. Introduction

On 30 January 2020, the novel coronavirus infection (COVID-19) was defined as a new Public Health Emergency of International Concern by the World Health Organization (WHO) [1], and as a pandemic on 11 March [2]. The virus soon spread around the world and nearly 164 million cases have been reported up until May 2021 [3]. Among them, 104,765 cases have been confirmed in China up until May 2021 [4] and 66% of them are located in Hubei Province, which accounts for the 4512 out of the 4857 Chinese deaths [5]. With 3.4 million deaths worldwide to date, a race against time has begun to produce effective vaccines to fight the disease [6]. 

After better understanding of SARS-CoV-2 transmission, several measurements, known as non-pharmaceutical interventions (NPIs), were taken to mitigate the spread of the virus and the government applied various regulations in different sectors such as tourism, transportation, etc. [7]. Moreover, similar to other countries, this pandemic has had profound financial and psychological impacts in China and it is important that these topics are addressed because they can be assessed as lessons for the possible future outbreaks or be considered as means of comparison between different countries.

On 10 January 2020, the full RNA genetic sequence of SARS-CoV-2 was released and published by Chinese researchers and then the world started to develop vaccines against this new disease [8]. Different means and platforms were used to develop vaccines, including protein subunits, viral vectors (non-replicating and replicating), DBA, RNA, inactivated virus, live attenuated virus, virus-like particles and antigen-presenting cells [9]. On 16 March 2020, an mRNA vaccine (mRNA-1273) developed by Moderna and an adenovirus type-5 vectored vaccine by CanSino started the phase I clinical trials [10,11]. In April 2020, three inactivated vaccines, i.e., Corona Vac (PiCoVacc) developed by Sinovac, BBIBP-CorV by Sinopharm/Beijing Institute of Biological Products and New Crown COVID-19 by Sinopharm/Wuhan Institute of Biological Products, and a DNA vaccine (INO-4800) entered clinical trials as well [12]. Additionally, to date, there are 112 vaccines in clinical development, as well as 184 vaccines in pre-clinical development [9]. 

Russian President Vladimir Putin announced the approval of a coronavirus vaccine developed by the Gamaleya Research Institute of Epidemiology and Microbiology in Moscow for widespread use on 11 August 2020 [13], and on 2 December 2020, the United Kingdom granted emergency use authorization for BNT162 developed by Pfizer and BioNTech [14], while WHO approved BNT162 as the first SARS-CoV-2 vaccine for emergency use on 31 December 2020 to encourage global manufacturing and distribution [15]. As of 7 May 2021, when a Chinese vaccine (BBIBP-CorV) was approved for emergency use by WHO, there were in total six vaccines with the approval from WHO [15]. China approved its first vaccine against SARS-CoV-2 for conditional marketing authorization on 30 December 2020 [16] and by May 2021 there were six Chinese vaccines granted with conditional marketing authorization or emergency use authorization. On the other hand, the emergency vaccination against this disease was started in July 2020 and the mass vaccination was formally launched on 15 December 2020 in China [16,17]. 

China was the first country hit by the pandemic, and it plays an important role in vaccine development and production. The present study is designed in order to have an overview of the situation of the outbreak and the vaccination strategy in China. It has been more than one year that the disease appeared and had profound impacts all around the world, and similarly China has been affected by this disease. At the time being, more data are available in a variety of fields, such as COVID-19 epidemiology, its psychological and economic impacts and also the management of the outbreak through decision-making on non-medical and medical interventions, mainly vaccinations.

Additionally, the authors believe that when there is a COVID-19 case study in a country it is essential to have a clear view towards the context of COVID-19 in that country, including demographic and epidemiological situation, the prevention plans, impacts of the media on how people follow the instructions and experience the pandemic era, and also the financial and psychological effects on the country regarding this situation. Moreover, in this paper, the Chinese vaccination process and strategy is discussed from the beginning and it gives a clear vision towards the attempts related to massive vaccination. Therefore, future studies can benefit from the present study, which provides data on the aforementioned aspects of COVID-19 in China. It is important to consider that this recent pandemic has had a serious impact on all the countries around the globe and still there is a great need to explore various aspects of the disease, such as vaccination strategy in different countries to examine its efficacy and the overall situation of a country based on non-pharmaceutical interventions and the media coverage. In this regard, the authors believe that this paper can promote background knowledge for further studies with similar aims.

## 2. Methodology

The present study is based on English, French and Chinese research to find relevant peer-reviewed articles, national and international databases and accessible information from the Chinese official websites and news agencies. The data on the number of COVID-19 cases were obtained from Statista [18], while the number of vaccine doses administered was accessed from Our World in Data [19]. The information about vaccination strategy in China was mainly from the website of the Press Conference of the Joint Prevention and Control Mechanism of the State Council. The clinical trial registration numbers or identifiers are available on ChiCTR and ClinicalTrials.gov.

With the aim of having the most comprehensive findings, the search for scientific articles was carried out through the following search engines, PubMed, Medline, Web of Science and Google Scholar, using the terms “COVID-19”, “SARS-CoV-2”, “epidemiology”, “vaccine”, “vaccination”, “case fatality rate”, “psychological impact”, “economic impact”, “media”, “social media”, “non-medical interventions” and “non-pharmaceutical interventions”.

In the result section, we explore and present the origins of novel coronavirus infection, sociodemographic characteristics of coronavirus disease cases, Chinese healthcare system and the current COVID-19 epidemiological situation in China. Then, the management and impacts of the new disease outbreak are analyzed, such as non-pharmaceutical interventions, economical and psychological impacts of the pandemic, and the influence of the media as well. After that, we continue to explore the vaccination strategy against SARS-CoV-2 in China, including the acceptance of vaccination, current available vaccines and the characteristics, national mass vaccination strategy, vaccine supply volume, vaccination procedure, and vaccine delivery outside China. In the discussion section, the results are briefly summarized and compared with other countries, as well as China’s role in developing and producing vaccines and the relevant criticism is discussed.

## 3. Results

### 3.1. Case Presentation

#### 3.1.1. The Origin of the Novel Coronavirus Infection

The novel coronavirus infection was first detected around 8 December 2019 in a 65-year-old man working in a market in Wuhan [20]. However, there is evidence that he was not patient zero, as a 55-year-old man would probably have been infected on 17 November 2019 [21]. Two scenarios could explain the origin of this novel coronavirus infection. 

Firstly, there is the so-called zoonotic transmission of SARS-CoV-2 from animals to humans [22] as animals and humans rub shoulders in Wuhan markets. Bats have been the number one suspects for a long time as original hosts because their coronavirus RaTG13 is very close to the SARS-CoV-2 [23], with more than 96% homology [22]. However, it is now clear that it is genetically not sufficiently similar to be qualified as the direct progenitor of COVID-19 because of “its spike diverges in the RBD, which suggests that it may not bind efficiently to human ACE2” [22]. Pangolins have then been suspected, because some of their coronaviruses “exhibit strong similarity to SARS-CoV-2 in the RBD, including all six key RBD residues”, but it is now clear that “no animal coronavirus has been identified that is sufficiently similar to have served as the direct progenitor of SARS-CoV-2” [22].

Secondly, there is the possibility that the SARS-CoV-2 was deliberately or accidentally spread from the Wuhan Institute of Virology, but there is still no evidence that corroborates this theory suggesting a human-made novel coronavirus [24]. Peter Ben Embarek, program manager at the WHO, explained that this scenario was extremely unlikely [25]. 

The most plausible scenario seems to be the first one, as increasing human interaction with wild animals and natural ecosystems is leading to the emergence of zoonotic pathogens that are likely to cause an outbreak such as the actual pandemic [23]. 

#### 3.1.2. Sociodemographic Characteristics of COVID-19 Cases

As the third largest country in the world after Russia and Canada, with a total surface area of 9.6 million square kilometers and the largest population in the world with more than 1.4 billion inhabitants, which represents 153 people per square kilometer, and a border with 14 countries [26], China decided to make strict decisions to contain the spread of the virus. The first COVID-19 lockdown started in Wuhan, the epicenter of the pandemic, on 23 January 2020, sealing off the city of 11 million people from the rest of the country [27]. 

Wuhan became the city with the highest number of cases in February 2020 according to a study on the disease surveillance data on symptomatic cases of COVID-19 conducted in 30 provinces in China between January and March 2020 [28]. These facts are consistent with the late lockdown measure that allowed the virus to spread across the city between November 2019 and January 2020. The virus then spread through the rest of the country in only one month and the previously cited study showed that urban living areas were more affected by the novel coronavirus infection than rural living areas, cumulating 6923 infections (54.7%) against 5724 (45.3%) [28]. This is not surprising as 59.7% of the Chinese population are living in urban areas, where viruses thrive because of high density and mobile populations [26]. The Chinese Center for Disease Control and Prevention (CCDC) collected 44,672 epidemiological data in February 2020 that showed that the majority of COVID-19 infections occurred in people aged 30 to 69 years old [29], and males accounted for 52% of the confirmed COVID-19 cases [28]. The median age of severe cases was 57 years against 43 years for the non-severe ones, and severe and critical cases were more likely to happen with aging and among males [28]. Of the 4857 deaths [5], 80% were attributed to patients aged 60 years old and above [29]. The Chinese overall fatality rate evolved between 3.1% on 22 January 2020 and 4.72% on 9 May 2021 [30], and varied across age categories, becoming more and more important with aging, and especially for people aged 80 and above. This relatively high percentage is probably due to the fact that China was the first country to be confronted with this unknown disease and that medical interventions took a certain time to be established. Moreover, it is now assumed that the fatality rate in China was underestimated [31] as the media and researchers have started to assume that the Chinese government was downplaying the severity of the crisis and hiding the real death toll [32] by excluding some infected people who died outside the hospital or without being tested and registered as dead due to COVID-19. 

Despite the pandemic, China was the only major economy to achieve a positive growth of 2.3% in 2020 [33]. This is not comparable with the 6.6% reaching point in 2018 but with a nominal GDP of $14.86 trillion in 2020 [34], the Chinese economy remains the second largest in the world and also one of the fastest growing economies of the 21st century.

#### 3.1.3. The Chinese Healthcare System

The Chinese healthcare system was highly centralized at its origin, and there were important rural/urban differences in resource allocation, leaving people with no health security [35]. Consequently, the health system has undergone several transformations and reforms since 1996. The first round of reforms did not resolve the lack of access to healthcare and the high out-of-pocket payments, as a large proportion of the population was still not able to afford the necessary healthcare [36]. The 1996 health system reform was thus qualified as a failure in 2005 and led to a new round of reforms in 2007. The Central Committee of the Communist Party of China and the State Council agreed on the “Healthy China 2030 Plan” to build an equitable and efficient health system for all, leading to more health security, better healthcare delivery and higher provision of essential medicines [36]. Subsequently, the universal coverage health insurance for urban and rural residents was created in 2009, decreasing the rate of out-of-pocket payments from 60% in 2001 to 34.4% in 2012 [37] and to 28.8% in 2016 [38].

To date, challenges and inequalities between urban and rural areas still exist despite all the progress. Prevention is somewhat still relegated to the background because of high investments in treatments. The health system has insufficient capacity to respond to various public health emergencies [39], as medical institutions are understaffed with 1.6 doctors per 1000 people in China and only 5.5% of the Chinese GDP work in the healthcare system [40]. Consequently, the Chinese health system is ranked 114th in the WHO classification [40].

The outbreak has put a strain on healthcare systems worldwide and China was not spared. Resources such as hospital beds or ventilators were limited, putting medical infrastructures under pressure. To remedy that situation, the Chinese authorities established 17 COVID-19 Specialty Ark Hospitals. This was a very effective solution to manage the Wuhan cases [41].

#### 3.1.4. Chinese Epidemiological Situation

Patients with unexplained pneumonia who did not respond to antibiotics treatments were detected in Wuhan in December 2019 [21]. Soon after that, many people developed similar symptoms and 55% of them had been at Wuhan’s Huanan wholesale seafood market, the place that is believed to be the epicenter of the epidemic [1]. At this point, the new outbreak was thus local and affected mostly people who had been at the market. However, in January 2020, many people without direct contacts at the market developed symptoms. The virus transmission had reached the dissemination phase within the community, prompting Chinese authorities to confirm that a novel coronavirus infection was definitely there. At this time, the basic reproduction number (R0) was 2.2, the average incubation period was 5.2 days, and only 7.4 days were needed to double the number of COVID-19 cases [1]. Additionally, 23.4% of infected patients developed critical consequences [1]. 

Soon after that, several COVID-19 clusters then appeared in Wuhan, and the movement of people for the Chinese New Year quickly spread the virus in the national and international community. For that reason, a strict lockdown was established in Wuhan on 23 January 2020, with different harsh measures to contain the virus spreading. Consequently, the WHO qualified this new coronavirus infection as a “Public Health Emergency of International Concern” on 30 January 2020, and named it “COVID-19” on 11 February 2020. Then, the “International Virus Classification Commission” officially talked about “SARS-CoV-2” to define this seventh type of coronavirus [1]. On 27 February 2020, Tedros Adhanom Ghebreyesus, Director-General of the WHO, asked all countries which still had no COVID-19 cases to prepare for the arrival of the virus because “no country should assume it won’t get cases; that could be a fatal mistake, quite literally” [42]. 

**Figure 1:**
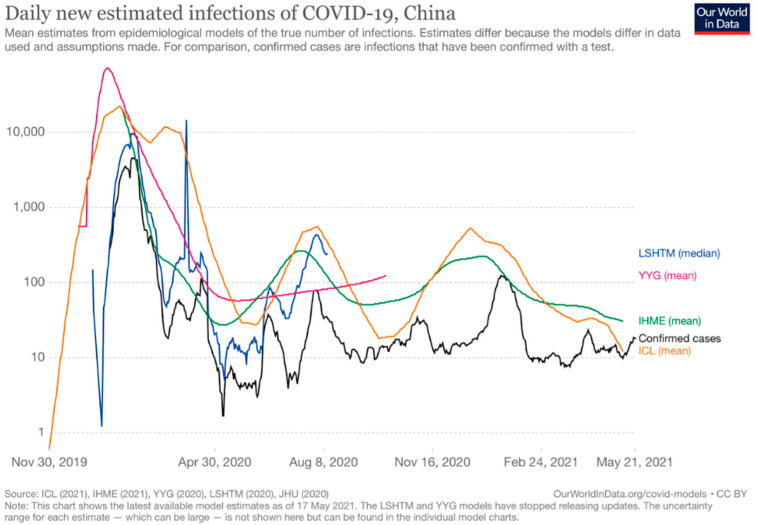
Daily new estimated infections of COVID-19 in China [43].

On 8 April 2020, the lockdown in Wuhan was lifted while the spread of the virus was growing worldwide. At this time, the Chinese fatality rate was stabilized at 5.7% [31], and the number of daily new estimated infections was decreasing as Figure 1 shows [43].

Since mid-2020, Sinovac and Sinopharm vaccines were locally approved for emergency vaccination before the end of the clinical trials period. Finally, in October 2020, the eastern Qingdao city officially started a mass testing campaign of nine million inhabitants after the emergence of a dozen COVID-19 cases [44]. The same happened in the city of Kashgar in Xinjiang province [45]. Strict lockdown, mass testing and vaccination are thus the main strategies adopted by China, which have been effective, as only 18 new confirmed infections were confirmed on 21 May 2021 [43].

### 3.2. Management and Impacts of the COVID-19 Outbreak

#### 3.2.1. Non-Pharmaceutical Interventions

Before having registered vaccines available worldwide, non-pharmaceutical interventions (NPIs) were almost the only approaches to prevent having the new emerging infectious disease. The effect of NPIs on its mitigation is a widely discussed subject among policymakers, economists, and research and clinical experts [7]. However, considering the shortage of vaccines, having no efficient treatment and also limited medical capacity, NPIs represented the only options to control the pandemic [46]. 

In January 2020, China implemented the strictest mitigation measures; so-called NPIs which had successfully controlled the virus’s spread by the end of February [47]. In response to the new outbreak in China, immediately the Central Leadership Group for Epidemic Response and the Joint Prevention and Control Mechanism of the State Council was formed and the Chairman Xi Jinping requested the prevention and control of the outbreak to be the top priority of the government [48]. These interventions and initiatives aim to minimize the spread, delaying the onset and reducing the size of the disease peak, buying time for healthcare system planning, and allowing the use of vaccines and medications in the future [47]. 

Based on the available studies, there are a varied range of NPIs and different categories for them and the following measures have been implemented in China since the early stages of the outbreak: obligation of face mask wearing in public places, quarantine for individuals with confirmed positive tests and people who had close contact, displacement of infected people, food distribution, physical distancing, traffic restrictions, closing schools, teleworking if possible, financial aids for the affected population, internal and international restrictions, contact tracing, cancelation of gatherings, hand washing and distributing the updated information via the media such as television and radio programs and newspapers and also social media [46,47,49,50,51]. 

Lai et al. indicated that if NPIs were not applied as of 29 February, the confirmed positive cases would significantly rise with a 51-fold increase in Wuhan and a 125-fold increase in other provinces of China, and if these measures could have been implemented one or two weeks earlier in the country, the number of cases could have been drastically decreased by 66% and 86%, respectively [47]. Thus, implementing the NPIs strictly at the initial months and maintaining the approach later is the key to the success of mitigating confirmed cases and controlling the spread of the virus [47,49,51].

Finally, taking the example of the city of Hangzhou can demonstrate the actual situation of COVID-19 management in China. In Hangzhou the NPIs were in three stages from 21 January to March 2020. In the first stage, which was from the date of the first confirmed case reported until 2 February, the interventions were quarantine of patients or suspected patients and also close contacts, and disinfection of locations where the patients had been previously, and a total of 109 confirmed cases were reported. The second stage was from 3 February to 18 February, when the government stepped up interventions after three districts announced cases from unknown origin on 3 February. Local transmission was clearly on the rise. Except for certain important places, such as supermarkets and hospitals, all businesses and institutions were closed. People were told to stay at home and were only allowed to go out twice a week to buy essentials. At this stage, 19 imported cases and 40 local cases had been registered, including nine cases from unknown origin. The third stage lasted from 19 February to early March. The outbreak was essentially under control after 15 days of containment. The containment order was lifted, and companies were free to resume normal operations. Every day, workers’ health status was to be monitored, and preventive disinfection of public areas was required at this point. As a result of this intensive control, no new case was identified as of 2 March [52].

#### 3.2.2. Impact of the Media

In dealing with a public health threat, crisis communication, which refers to effective and precise communication to a variety of people during emergency situations, is critical [53]. In case of emerging infectious diseases, the risks and uncertainties can arouse public emotions and change behaviors in either positive or negative ways [54]. Additionally, understanding how important information about a health threat is disseminated, as well as how the public accesses and uses this information, is essential during an outbreak [55]. There is a belief that the media can be the primary means for health interventions, especially in emergency settings and infectious outbreaks [56] based on the previous studies which were carried out during the past public health crisis such as H1N1 influenza [57,58,59].

Generally, in China, central government media, namely CCTV, People’s Daily, and Xinhua News Agency pay more attention to news about national policies and the state’s and leadership’s image. Local news agendas, on the other hand, are set to and interested in local interests [53]. However, during the pandemic in China, generally the media, at governmental and local levels, was a significant source of information during the COVID-19 outbreak, specifically in the first stage of the outbreak [60] as the authorities issued regular COVID-19 updates via news media coverage in order to raise awareness about the disease and recent NPIs [53].

Considering the beginning of the outbreak, the first news about the new virus was released on 31 December 2019, indicating 27 pneumonia cases were confirmed in Wuhan [56]. Liu et al. found out that the media coverage in China prevented 394,000 additional new cases and 1.4 million close contacts from 19 January to 29 February [56]. Additionally, a study in Hubei province shows that if the amount of information in the media decreases, the number of cases would significantly rise [61]. This may be as a result of people’s great trust and reliability on the governmental media for health-related information and the fact that they followed the advised interventions during the pandemic [53].

Focusing on social media and the use of mobile applications, China is trying to be technologically independent in this field by dedicating 2.1% of GDP to research and development. Of this budget, 77% goes to high-tech companies and therefore a number of applications are growing [62]. Having said that, social media was one of the important means of the disease management, specifically through nation-wide applications such as Weibo and WeChat. WeChat has more than one billion Chinese users and offers a variety of services such as messaging, and posting news or documents. Thus, people easily can follow the related news and be under the influence of the media content [63].

#### 3.2.3. Economic Impact

China has made remarkable progress in economic growth after the reforms began in 1978 [64]. The global economy began to decline after the financial crisis of 2008, but China’s economy continued to expand at a moderate pace [65]. As a result of the novel coronavirus presence in 2019, worldwide limitations such as travel bans and closing borders are wreaking havoc on the global economy and financial markets in ways that have not been seen since World War II [66] and China is not an exception in this issue [67].

The economy of China is interconnected with other countries in the fields of business, tourism and investment and therefore any recession or continued transportation restrictions would likely put pressure on global supply chains [65]. In addition to the global contacts, more than 18 million enterprises operate inside of China, accounting for nearly 80% of employment and 50% of private sector exports. The coronavirus epidemic has had a significant impact on these companies, specifically in the first couple of months [65].

The measures undertaken in order to control the spread of the virus had a profound impact on the agriculture economy, which is one of the essential bases of the economy [68]. In the first four months of the COVID-19 outbreak, China faced a 6.8% drop in GDP compared to the same period in 2019, with the agri-food system losing 7% of its value added, which is equal to almost RMB 0.26 trillion, and about 46 million agriculture field workers lost their jobs [48].

As discussed before, travel restrictions at all levels had an immediate impact on national economies, including the tourism industry, which has suffered profoundly, through the loss of income from overseas travel, national tourism and infrastructures such as accommodation, public transportation, air lines, boats and cruises and entertainment places, for instance restaurants [66,69]. According to the United Nations World Tourism Organization (UNWTO), major tourist spots had adopted travel restrictions in response to the pandemic by 20 April 2020 [69].

In China as well, the tourism industry is an important sector of the economy which brings in around RMB 5128 billion per year for the country. However, the industry has been severely impacted by the presence of SARS-CoV-2 [70,71]. From 20 April 2021, China began to accept those seeking to enter the country who were vaccinated in the United States with COVID-19 vaccines manufactured by the US pharmaceutical companies. Before this time, only individuals who had been inoculated with Chinese vaccines were allowed to enter the country [72]. At the moment, travel restrictions are still implemented including 14 days of quarantine after abroad arrival and no entry permits to foreign students [73]. However, it is estimated that there will be an increase in the industry, especially in domestic tourism which will be more than four billion trips inside of China, meaning that internal tourism will bring RMB 3.3 trillion in revenue in 2021 [74].

With the COVID-19 outbreak effectively under control, the Chinese government has started to implement various measures and has been gradually but steadily resuming its economy since early March 2020 [48,65,67]. It must be considered that the pandemic is not over yet and there are still measures to control the spread of COVID-19 in China and around the world, therefore, the impacts on the different sectors of the economy are ongoing through all the affected countries [48].

#### 3.2.4. Psychological Impacts

The COVID-19 outbreak has led to significant mental health concerns including stress, insomnia, anxiety and depression [75,76,77]. Different groups of the population within a society can face psychological issues due to the pandemic. The patients might be afraid of mortality or late physical consequences of the disease. Healthcare personnel who are in contact with these patients may be worried about catching the disease or transmitting the virus to their loved ones [76,78]. People who have lost a family member or a friend experience the grief, sadness and anxiety [79,80]. Staying in obligatory quarantine can be a stressful encounter [81]. Patients with underlying physical and/ or mental health conditions might face troubles to access medical care, so this situation might deteriorate their health conditions and this can increase the stress level [82]. Such temporary experiences might lead to long-lasting mental health conditions such as post-traumatic stress disorder, adjustment and/or anxiety disorders [78].

Regarding alcohol consumption, it has been proved that it is positively associated with depressed mood and anxiety [83]. Additionally, having fewer social contacts can contribute to the increase in drinking alcoholic beverages [84]. It was estimated that excessive alcohol consumption in China was about 4.4% of the population in 2018, while the number has increased during the pandemic to 11.1% [78]. 

As China had suffered from the SARS epidemic in 2003, the decision-makers could benefit from that experience. Therefore, on 26 January 2020, the National Health Commission published a guidance outlining the principles for emergency psychological crisis strategies to mitigate the psychosocial impact of the outbreak [85]. This guideline, “Principles for Emergency Psychological Crisis Intervention for COVID-19 Pneumonia Epidemic” asks mental health organizations and universities to collaborate and support the online platforms in large scales as a promising substitute for in-person consultations [78]. Additionally, a number of videos were produced in order to raise people’s awareness about mental health and to provide practical instructions through the common social media, WeChat, and other Internet-based platforms. All in all, from 26 January to 20 February 2020, 29 guidelines and instructions were released in order to protect mental health in different groups of people [82].

Although China had begun to protect mental health at the early stage, the efficacy of psychological services was not clear at the time of the crisis. Additionally, since online psychological services are the most common form of assistance, certain people, namely elderly people, with restricted access to the Internet and smart devices, may not be able to use such services properly [82]. Moreover, the number of studies on mental health at the first stage of the pandemic was considerable, but it is important to continue psychological interventions and the research in this field in order to assess the interventions. Additionally, the psychological impact of this pandemic might be long-lasting, similar to the impact in the years following the SARS outbreak. A study in Hong Kong showed that 42.5% of SARS survivors indicated at least one mental health condition and the most repeated ones were post-traumatic stress disorder and depression, respectively [86]. Another limitation in the field of mental health during the COVID-19 is that Chinese scholars publish their findings in English-language journals. Thus, as a result of the language barrier, local health providers and decision-makers may not be able to learn from these results [82].

### 3.3. Vaccination Strategy

#### 3.3.1. Acceptance of COVID-19 Vaccination in China

Vaccination is considered as a critical strategy against emerging infectious diseases, and COVID-19 is no exception [87]. However, the availability of vaccines does not guarantee the uptake of vaccination in the population in reality. A typical case is the vaccination against H1N1. Although vaccine against the pandemic (H1N1) 2009 was developed rapidly thanks to the influenza vaccine development technology and the platform was relatively matured [88], the willingness of people to accept the pandemic (H1N1) 2009 vaccine among the general public differed from 8% to 67% across ten studies [89], while the vaccination coverage ranged from 0.4% to 59% across 22 European countries [90]. Vaccine hesitancy is common, and it reflects people’s perception of the disease risk and the attitude to vaccines [91,92]. Therefore, it is of importance to understand the acceptance of vaccination against the disease among the population, in order to reduce people’s hesitance and promote the population vaccination to achieve a certain level of herd immunization against COVID-19 [93]. 

In this section, the acceptance of SARS-CoV-2 vaccination in China will be assessed thought two cross-sectional surveys which were conducted during the pandemic period (severe phase) and post-pandemic period (well-contained phase) of the coronavirus disease c in China by Wang et al. [94,95]. The comparison of the acceptance of the COVID-19 vaccination between the two surveys is presented in Table 1.

The first survey was conducted in March 2020, when China was still in the severe COVID-19 pandemic phase and vaccines were still under development. The results indicated that the majority (91.3%) of the participants showed an intention to receive COVID-19 vaccination if the vaccines were developed successfully, among which 52.2% stated that they hoped to be vaccinated as soon as possible when the vaccines were available, while others (47.8%) expressed that they would delay the vaccination until the safety of the vaccines was confirmed [94]. The second survey was conducted between November and December 2020, when the pandemic was well contained and the vaccine would soon be available in China. Among the 791 respondents longitudinally followed up in two surveys, the percentage of people who wanted to be vaccinated dropped from 91.9% to 88.6%, and in the vaccine acceptance group, the percentage of people who wanted to receive vaccination as soon as possible when vaccines were available declined from 58.3% during the severe phase to only 23.0% during the well-contained phase; even among all the respondents in the second survey, fewer people (88.5%) expressed a desire to be vaccinated, among which 24.7% wanted to be vaccinated immediately [95].

From the two studies, the results demonstrated that the willingness to accept COVID-19 vaccination among the general population in China slightly decreased from the severe phase to the well-contained phase; however, the willingness to receive immediate vaccination reduced dramatically. The surveys also suggested that concerns about vaccine safety, convenience and accessibility of vaccines were the main barriers for mass vaccination to be rolled out. Therefore, the vaccination strategy ought to be designed to remove these barriers in order to increase the vaccination coverage. Responsible and reliable information dissemination about vaccine safety, efficacy, effective health education and vaccination convenience could be considered when the authorities design the mass vaccination strategy [94,95].

#### 3.3.2. Available Vaccines and the Characteristics

##### The General Vaccine Development and Approval Process

According to the Vaccine Administrative Law of the People’s Republic of China, which was adopted by the Standing Committee of the Thirteenth National People’s Congress of the People’s Republic of China on 29 June 2019, and which came into force on 1 December 2019, and the relative technical guidance [96,97,98], clinical trials could only be conducted after the approval by National Medical Products Administration of the State Council (NMPA). Additionally, the clinical trials are to be organized by tertiary medical institutions qualified by NMPA and the health authority of State Council or Disease Prevention and Control Institutions at or above the provincial level, while NMPA is responsible for the supervision and administration of vaccines nationwide [96].

Vaccines that are urgently needed for disease prevention and control and innovative vaccines could be prioritized during the review and approval procedure by NMPA. When facing any particularly severe public health emergency or any other emergencies that seriously threaten public health, the health authority of State Council could propose for emergency use of vaccine in accordance with the needs of the prevention and control of the disease, and the vaccine would be assessed and then approved by NMPA for emergency use with a certain time and scope limit; and if the benefits outweigh risks upon assessment and the protection efficacy should reach at least ~50% (point estimate) with the lower limit of the 95% confidence interval not less than 30% while over 70% (point estimate) of efficacy is preferred in accordance with the requirements of NMPA Guideline for Clinical Evaluation of Novel Coronavirus Preventive Vaccines (Interim) and WHO Target Product Profiles for COVID-19 Vaccines [97,99], the vaccine could be evaluated and then granted conditionally marketing authorization by NMPA [96]. After being approved for conditionally marketing authorization, the following work is still required to be carried out for the vaccine: (1) in the case of using the interim analysis data of clinical trials, completing the phase III clinical trial is required; (2) in the case of using overseas clinical trial data, it’s required to conduct domestic clinical researches in accordance with relevant requirements [97].

##### Current Available Vaccines

In June 2020, China approved the emergency use of three Chinese vaccines against SARS-CoV-2 in accordance with law [17]. As of May 2021, there were six available vaccines granted conditional marketing authorization or emergency use authorization: four inactivated vaccines (BBIBP-CorV, Cornona Vac (PiCoVacc), New Crown COVID-19 and KCONVAC), one viral vector vaccine (Ad5-nCoV) and one recombinant protein subunit vaccine (ZF2001), as shown in Table 2. 

For the inactivated vaccines, the virus was cultured and proliferated in Vero cells. β-propionolactone was used to inactivate the virus while the antigen components were retained in order to induce the immune response of the body, and aluminum hydroxide adjuvant was utilized to improve the immunogenicity [104]. Among the four inactivated vaccines, BBIBP-CorV, which was developed by Beijing Institute of Biological Products/Sinopharm, was granted conditional marketing authorization on 30 December 2020 by NMPA; and Corona Vac developed by Sinovac Biotech was granted conditional marketing authorization on 5 February 2021, while New Crown COVID-19 developed by Wuhan Institute of Biological Products/Sinopharm were conditionally licensed for marketing on 25 February 2021 [105]. Additionally, on 7 May 2021, KCONVAC developed by Shenzhen Kangtai Biological Products and Beijing Minhai Biotechnology was approved for emergency use [106].

To develop the adenovirus type-5 vectored vaccine, the genes of the spike glycoprotein of SARS-CoV-2 was recombined into the genes of replication defective Ad5, then the genetically recombined adenovirus expressed the spike glycoprotein of SARS-CoV-2 and thus induced the immune response [104]. Ad5-nCoV was developed by CanSino Biologics Inc. and Academy of Military Medical Sciences, PLA of China, and was approved for conditional marketing on 25 February 2021 [105]. ZF2001 developed by Anhui Zhifei Longcom Biopharmaceutical was approved for emergency use on 10 March 2021 [107]. To develop this vaccine, the genes of the receptor-binding domain (RBD) of spike protein were recombined into the genes of Chinese hamster ovary (CHO) cells, and then the genetically recombined CHO cells expressed RBD dimer in vitro; additionally, aluminum hydroxide adjuvant was used to improve the immunogenicity [104].

Before the phase III trial data of Chinese COVID-19 vaccines were published in the peer-reviewed journals, the relevant data were only shared through announcements from the manufacturers and the governments in countries where the trials were conducted, or based on interim phase III data [102]. For example, the efficacy of Corona Vac developed by Sinovac ranges from 50.4% to 86% in different countries, while Ad5-nCoV was reported with an efficacy of 65% for preventing symptoms and 90% for preventing severe symptoms. The efficacies of these Chinese vaccines are considered relatively lower compared with those developed in Europe and the US. Additionally, some researches have raised concerns of lack of transparency of Chinese vaccines [108]. On 26 May 2021, the phase III trial data of two inactivated SARS-CoV-2 vaccines developed by Sinopharm were published, stating an efficacy of 78.1% for BBIBP-CorV and 72.8% for New Crown COVID-19 [101].

However, the efficacy of the current vaccines against B.1.617.2 (delta) infection requires more research. A case–control study conducted in Guangzhou, China estimated that the vaccine efficacy of Chinese inactivated SARS-CoV-2 vaccines was 59.0% (95% CI: 16.0% to 81.6%) against the delta variant after two-dose full vaccination, which is lower compared with the efficacy reported before [101,109]. The efficacy also seems to be lower in comparison with BNT162b2 and ChAdOx1 nCoV-19 vaccines, whose efficacy was estimated to be 88.0% (95% CI, 85.3 to 90.1) and 67.0% (95% CI, 61.3 to 71.8) among persons with the delta variant, irrespectively [110].

There are also concerns about the adverse events after vaccination. The monitoring information on adverse events of SARS-CoV-2 vaccination in China from 15 December 2020 to 30 April 2021 was released by CCDC. The incidence of adverse effects after vaccination was reported to be 11.86/100,000 doses. Among 0.265 billion doses administered, 31,434 cases of adverse reactions were reported, among which 26,078 cases (9.84/100,000 doses) were common adverse events, such as fever (≥38.6 °C, 2722 cases), redness and swelling at the injection site (diameter ≥ 2.6 cm, 675 cases) and induration (diameter ≥ 2.6 cm, 304), representing 82.96% of total adverse events; on the other hand, 5356 uncommon adverse events were also reported (2.02/100,000 doses) including allergic rash (3920 cases), angioedema (107 cases), and severe allergic reaction (75 cases) [111].

A national cross-sectional survey conducted in Turkey indicated that 62.5% participants reported that they experienced at least one adverse effect after receiving Corona Vac [112]. Localized pain at the injection site was the most common side effect (41.5%), followed by fatigue (23.6%), headache (18.7%), muscle pain (11.2%), joint pain (5.9%), and nausea (5.3%) [112]. Another study among healthcare workers in China found that the incidences of adverse reactions of Corona Vac vaccine were 15.6% and 14.6% for the first and second dose, respectively, with the most common reaction being pain at the rejection site (9.6%), and other systemic adverse reactions, such as fatigue (8.3%), muscle pain (8.1%), headache (6.0%) and fever (2.9%) [113]. The adverse effects following Sinopharm COVID-19 vaccination seemed to be mild and predictable in United Arab Emirates, and pain at the vaccination site, fatigue and headache were the most frequent sides effects [114].

#### 3.3.3. National Vaccination Strategy

##### “Three-Steps” and “Two-Emphases” Vaccination Strategy and Prioritization of Target Groups

In July 2020, China officially launched the emergency use of vaccines against the coronavirus disease 2019 [17]. On 15 December 2020, the COVID-19 vaccination program among key groups, i.e., individuals who face a higher risk of exposure to SARS-CoV-2, including health workers, those who work to maintain production and daily supply of energy, water, food and transportation, and those who travel abroad to study or work in higher-risk countries, was formally launched; by 31 December 2020, within half a month, more than three million doses were administered among key groups [16].

According to the State Council Interagency Task Force, in order to reach herd immunity against COVID-19 through vaccination, a “three-steps” vaccination strategy was officially launched on 15 December 2020, starting with vaccination for the key groups [16]. The population was divided into three priority groups: (1) key groups: people who face a higher risk of occupational exposure including health workers and community workers, those who are at risk from overseas infection such as people working in airports; those in essential positions of maintaining the basic operation of society including workers engaged in maintaining national security, and production and daily essential supply; and those who are over 18 years old and live in border counties; (2) high-risk groups: elderly people and people with underlying diseases; (3) the general public; the first two groups were prioritized and young people below 18 years old were not considered at this time because more data are needed for the safety and efficacy of the current available vaccines for this group [16,105].

The “three-steps” strategy is in accordance with the three priority groups. At the first step, key groups will be vaccinated in order to maintain the function of society. Then, the second step is to vaccinate high-risk groups, as elderly people and people with underlying diseases are more likely to suffer severe symptoms after contracting coronavirus infection. In the third stage, vaccination is rolled out to the general public. The ultimate goal is to effectively protect the whole population through phased vaccination [16,105].

In the meantime, following the “three steps” strategy and in accordance with China’s approach of preventing the coronavirus from entering the country and stemming its domestic resurgence, currently, China is promoting the vaccination mainly focusing at “two key emphases”: (1) key areas: high-risk cities and areas are prioritized for vaccination, which include port cities, borders areas, large and medium-sized cities and areas where clustering cases of COVID-19 have occurred; (2) key populations: health workers, personnel from government agencies, enterprises and institutions, students and staff from higher education institutions, people who work in large supermarkets and those in transportation and logistics sectors to maintain the operation of the society, and people working in welfare institutions are also prioritized for the vaccination [115].

Yang and colleagues estimated the size of the target population of different priority groups. 49.7 million was estimated for the population of key groups. If the vaccination was extended to high-risk groups, 563.6 million people were estimated as needing to be vaccinated, while when the vaccination reaches the general population, an additional 784.8 million people would be in demand for vaccines [116]. Therefore, China is dealing with a great pressure in terms of producing vaccines, considering its large population.

##### Vaccine Supply Volume and Its Dynamics

In order to accelerate the industrialization of SARS-CoV-2 vaccines, a special work team was set up by the Ministry of Industry and Information Technology of the People’s Republic of China (MIIT) to ensure smoothing production of the vaccines. The “daily reporting, weekly scheduling and semi-monthly announcement” working mechanism was developed, while a monthly dynamic adjustment mechanism was also established [16,105]. Additionally, the whole industry and supply chain is reviewed by MIIT, and enterprises are required and guided to perform analysis on the supply risks of the key raw materials, equipment and consumables, as so to ensure the stability of the production and supply chain of COVID-19 vaccines [16].

By end of 2020, 18 Chinese domestic enterprises had successively started the production capacity building according to the progress of SARS-CoV-2 vaccine research and development, among which, Beijing Biological Products Institute and Wuhan Biological Products Institute of Sinopharm, and Sinovac accomplished the production capacity building task for the year of 2020 [16]. Production capacity building is a dynamic and continuous process, which would be promoted and progressed in accordance with the demand. Additionally, the large-scale production of vaccines could be launched rapidly when they are licensed for marketing due to the building of production capacity before the vaccines are granted marketing authorization. For example, after BBIBP-CorV was granted conditional marketing authorization, Beijing Biological Products Institute of Sinopharm has launched the large-scale production program immediately [16].

The production capability of vaccines is still being expanded, and the output is increasing accordingly. The output of the Sinopharm vaccine is projected to be more than one billion per year, and Sinovac has fulfilled the basic requirements of the production capacity of one billion per year; meanwhile, the biological products institutes of Sinopharm in Changchun, Lanzhou, Chengdu and Shanghai have also contributed to expanding the production capacity [117,118]. Additionally, the projected production capacity of KCONVAC by Shenzhen Kangtai Biological Products is 100 million per year [119]. It seems as though China will not face too much pressure of vaccine production, but the fact that China is also delivering vaccines to other countries cannot be ignored, which would increase the burden of vaccine production. Although the domestic demand of vaccines is prioritized, some people reported experiencing difficulties in having the second dose of the COVID-19 vaccine [105,120]. When scheduling the deployment of vaccines, it is of significance that the command of the second dose should be prioritized.

##### Vaccination Procedure for Individuals

**Figure 2:**
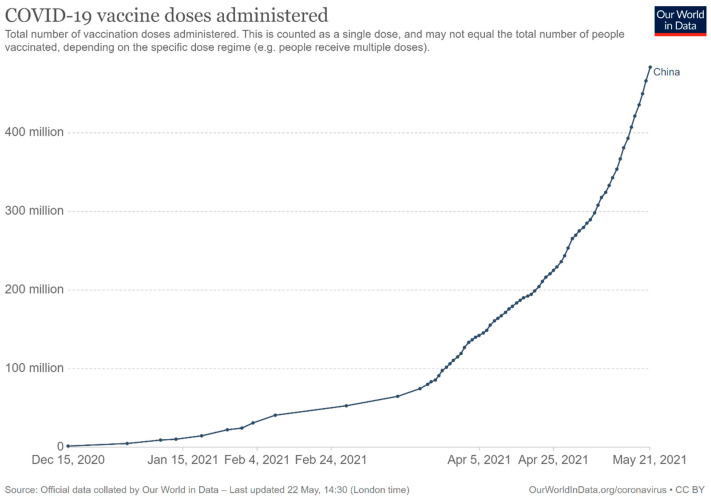
The number of COVID-19 vaccine doses administered in China [19].

**Figure 3:**
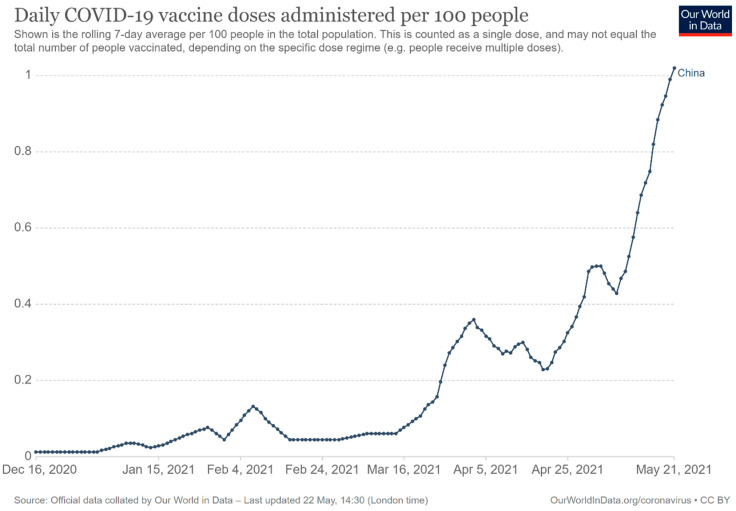
Daily COVID-19 vaccine doses administered per 100 people in China [19].

Since the mass vaccination program was formally launched on 15 December 2020, as of 21 May 2021, 483.34 million doses had been administered across the country [19]. Figure 2 shows the number of COVID-19 vaccines doses administered in China from 15 December 2020 to 21 May 2021, while Figure 3 indicates daily vaccine doses administered per 100 people in China. On 29 March 2021, the National Health Commission of the People’s Republic of China issued the Technical Guideline for the Inoculation of SARS-CoV-2 Vaccines to better guide health facilities at all levels to carry out the vaccination [104]. There are mainly three ways for people who are willing to receive the vaccination: (1) go to the health facilities, including healthcare centers, hospitals and centers for disease control and prevention at different levels, directly; (2) go to temporary vaccination sites directly; (3) online appointment and then go to the health facilities.

Before vaccination, the health status of vaccine recipients will be checked, such as whether they have underlying diseases, whether they are during the onset of diseases, and whether they have a history of allergies. After vaccination, recipients shall stay at the vaccination sites for 30 min in case some adverse event happens, and if any adverse event occurs, health workers are able to deal with it immediately. On the other hand, if no symptom related to adverse reactions occurs, the recipients may leave the sites.

For non-Chinese citizens who are 18 years old and above, the vaccination procedure is similar and vaccination is available in Beijing, Shanghai, Tianjin, Zhejiang province and Guangdong province [121].

Vaccines are free for Chinese citizens and no fees will be charged for the services. The vaccination is free for non-Chinese citizens who have joined China’s social medical insurance, while around RMB 100, which is around USD 15.45 with an exchange rate of 6.47, will be charged per dose for those who do not have [121].

##### Clinical Management of Potential Adverse Events

There might be some common adverse reactions after COVID-19 vaccination, such as headache, slight fever, redness at the injection site, for some other people, there might be cough, loss of appetite, vomiting, and diarrhea [122]. However, all health personnel engaged in the vaccination are trained professionally and strictly and at every vaccination site, experienced health workers for emergency treatment are deployed, together with necessary medical equipment and medicines, in order to identify suspected adverse events following vaccination and to treat them timely and professionally. Additionally, ambulances are also required to be stationed on sites [105].

#### 3.3.4. Vaccine Delivery Outside China

COVAX is initiated and co-led by WHO, the Vaccine Alliance (Gavi) and the Coalition for Epidemic Preparedness Innovations (CEPI), which aims to promote the equitable access to COVID-19 vaccines and deliver two billion doses by the end of 2021, in order to protect the frontline healthcare workers and the most vulnerable groups [123]. China officially joined COVAX on 9 October 2020 and stated the same goal as the COVAX facility to “promote international cooperation in COVID-19 vaccine research and development and favor developing countries in vaccine supply” [124].

**Figure 4:**
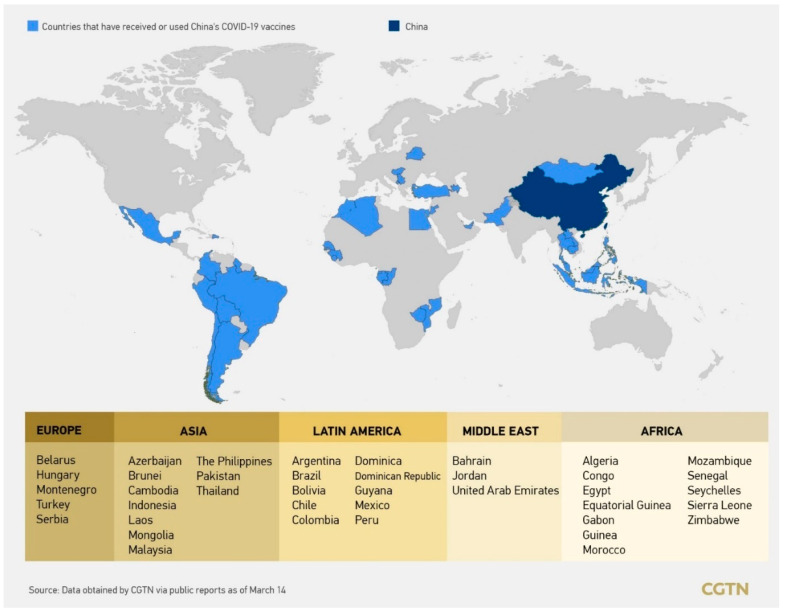
Countries that have received or used China’s COVID-19 vaccines by 14 March 2021 [126].

Five vaccines developed in Europe and the US have been approved for emergency use by WHO, in addition to which, one Chinese inactivated vaccine developed by the Beijing Institute of Biological Products of Sinopharm was approved by WHO on 7 May 2021, while another Chinese vaccine developed by Sinovac is still in process of the emergency use evaluation from WHO [15,125]. However, before this Sinopharm vaccine was listed for emergency use by WHO, Chinese vaccines had been authorized for use among more than 60 countries, and the vaccines are being donated to 69 developing countries and exporting to 43 countries as of 7 March 2021 [126], and 240 million doses have been delivered outside China, while another 500 million doses were committed to be shipped out [127]. Figure 4 demonstrates the countries that have received or used Chinese SARS-CoV-2 vaccines by 14 March 2021. Countries in Africa and Latin America are preferring to choose Chinese, Indian and Russian vaccines; at the same time, it is difficult for vaccines developed by the West to be reached by developing countries, who are under the pressure of purchasing them from wealthy countries who have secured at least 70% doses of the former five vaccines approved by WHO—both through COVAX and mainly via additional purchase contracts with developers for their population—which accounts for 16% of the global population [100]. China has stated that COVID-19 vaccines developed by China will be provided as a global public good and be accessible and affordable at a fair and reasonable price with developing countries being prioritized for the distribution of Chinese vaccines [128], whereas the price of vaccines is not relatively low [15]. On the other hand, some researchers criticized that China is acting to promote its good image and boost its influence among developing countries by delivering vaccines, as the case of supplying and donating medical products to countries during the first wave of the pandemic [129].

## 4. Discussion

In this case study, the country of China has been assessed from the viewpoint of the general coronavirus disease situation, management of the outbreak and possible impacts and the vaccination strategy. Considering the fact that China is one of the COVID-19 vaccine producers, it is important to understand its plans and strategy inside and outside of the country. From the perspective of economy management, the study found that although the country was hit hard by the disease, it could have a low positive rate of economic growth. Additionally, the media could deliver adequate information on the disease and non-pharmaceutical interventions that were applied were effective, as the disease was controlled in the first quarter of 2020. The mass vaccination against SARS-CoV-2 was formally launched on 15 December 2020 following the “three-steps” and “two-emphases” strategy, and has progressed steadily with 483.34 million doses having been administered across the country as of 21 May 2021 [19].

As several SARS-CoV-2 vaccines are available, the world has started to control the pandemic with vaccination. China formally started its national mass vaccination on 15 December 2020, and doses have been administered gradually since December 2020 and now steadily, with several millions being rolled out every day and 483.34 million doses administered as of 21 May 2021 [19]. “Three-steps” and “two-emphases” vaccination strategies are followed. Key groups including healthcare workers and other workers who are essential to maintaining the function of society are the first priority to receive COVID-19 vaccination, followed by high-risk groups (i.e., elderly population and people with underlying diseases) and then the general public. Similar strategies could be seen in Israel and the UK, although care home residents and their care workers are prioritized before healthcare workers and social care workers, followed by different age groups in order with elder groups being prioritized in the UK [130,131]. However, in Indonesia, the younger population aged 18–59 is prioritized after vaccinating healthcare workers and other frontline workers, where it is considered that the younger population are more likely to contract the new coronavirus infection and more information is needed for the safety and efficacy of vaccines for elderly people [132].

By 10 May 2021, 59.2 million doses of COVID-19 vaccines have been shipped through COVAX to 121 countries across the world, whereas COVAX aims to deliver two billion doses [15,123]. Meanwhile, as one of the biggest producers of vaccines against SARS-CoV-2 to the world, India delayed the shipment of vaccines outside the country and shifted the supply to its domestic demand, due to its struggle facing the current raging outbreak of COVID-19, while the US is greatly progressing its domestic vaccination at the cost of global supply, which were considered as one of the reasons that have driven countries to turn to China for vaccines supplies [127]. Additionally, by supplying vaccines to developing countries, China has faced criticism, with claims that China is providing vaccines in order to gain political image and influence [129].

China plays an important role in developing and producing SARS-CoV-2 vaccines, which would improve the global development and production capacity of the vaccines and the access to vaccines for un-wealthy countries. There is no doubt that China supplying vaccines to other countries will bring these following benefits: assisting countries to combat the coronavirus disease through vaccination, economic gains, increasing China’s good image and influence, and promoting China’s political friendship with other countries. However, which benefit China is prioritizing by supplying the vaccines to developing countries depends on individual’s perception. Additionally, should the perception be based on political perspective? As diseases do not distinguish people and countries politically, the vaccines should not be politicized either by suppliers, donors and purchasers, recipients or other indirectly related countries, and should only be viewed in terms of health.

On the other hand, the approval from WHO on the emergency use of a Chinese vaccine developed by Sinopharm could broaden the availability of COVID-19 vaccines and might facilitate the access of vaccines to developing countries, either via COVAX or contracts between countries and developers [125,127]. However, how many vaccines China could provide depends on its production capacity, which is dynamic and could be improved. Meanwhile, Egypt signed an agreement with Sinovac to produce the Sinovac COVID-19 vaccine, which is hoped to support the supply of vaccines both for Egypt and in African countries [133,134]. Additionally, India expressed its confidence in resuming its supply of vaccines to the world once the current outbreak is under control [127]. We hope either China or any other countries which have the capacity to manufacture safe and effective SARS-CoV-2 vaccines could further promote the production capacity to not only contribute to meeting the domestic demand but the international need as well.

The study also faces some limitations. First, the prevalence and fatality rate of the coronavirus disease cases might be underestimated, as at the beginning of the pandemic some cases who died outside the hospital or without being tested and registered might not be included. Additionally, the data of the vaccine production volume might not be 100% accurate due to the lack of information and transparency on it, and the production volume is dynamic and could be changed and even improved according to the time and needs. Another limitation is that the information about the vaccination strategy in China was mainly obtained from the website of the Press Conference of the Joint Prevention and Control Mechanism of the State Council, which is in Chinese; however, we have an author who is from China, so the language was not a problem for conducting the study. Additionally, the fact that Corona Vac developed by Sinovac was approved for emergency use by WHO on 1 June 2021 is not mentioned in the study as the main content of the paper was finished on 21 May 2021.

## 5. Conclusions

Vaccination is almost the only approach to end the pandemic in the world; therefore, it is vital to inspect each countries’ vaccination strategy to make efforts more efficiently for eliminating the disease. In this paper, China vaccination strategy is explored through assessing people’s acceptance at the beginning of the pandemic, when the acceptance was high, and approximately a year after when there was less demand from the population. In addition to that, Chinese vaccines and their features are discussed and it is found that until 21 May 2021, there were six vaccines available for mass production, with two of them having WHO authorization. Regarding the national delivery of the vaccines, “Three-steps” and “two-emphases” and relative registered doses are explained. As a COVID-19 vaccine-producing country, its contribution to providing vaccines for other countries, specifically low- and middle-income countries, has been recognized. All in all, it is important to look at various aspects of the current global health issue, the COVID-19 pandemic, including vaccine strategies in different countries and global efforts are required to ensure safety and health for everyone.

## Figures and Tables

**Table 1:** The comparison of the acceptance of the COVID-19 vaccination between two consecutive surveys in severe epidemic phase (March 2020) and the well-contained phase (November–December 2020) [95].

	Longitudinal Samples			Cross-sectional Samples		
	March 2020 (Severe Epidemic Phase)	November–December 2020 (Well-Contained Phase)		March 2020 (Severe Epidemic Phase)	November–December 2020 (Well-Contained Phase)	
	N (%)	N (%)	*p* Value	N (%)	N (%)	*p* Value
Overall respondents	791 (100)	791 (100)		2058 (100)	2013 (100)	
COVID-19 vaccination is an effective way to prevent and control COVID-19						
Yes	718 (90.8)	746 (94.3)		1842 (89.5)	1874 (93.1)	
No	73 (9.2)	45 (5.7)	0.007	216 (10.5)	139 (6.9)	<0.001
Accept vaccination if the COVID-19 vaccine is successfully developed and approved for listing in the future						
Yes	727 (91.9)	701 (88.6)		1879 (91.3)	1782 (88.5)	
No	64 (8.1)	90 (11.4)	0.03	179 (8.7)	231 (11.5)	0.003
Vaccine accept group	727 (100)	701 (100)		1879 (100)	1782 (100)	
Want to receive vaccination as soon as possible when the vaccine is available						
Yes, as soon as possible	424 (58.3)	161 (23.0)		980 (52.2)	441 (24.7)	
No, delay vaccination until I confirm the vaccine’s safety	303 (41.7)	540 (77.0)	<0.001	899 (47.8)	1341 (75.3)	<0.001

**Table 2:** Current available vaccines in China by 21 May 2021.

Vaccine	Type	Developer	Registration Number/Identifier (Country of Recruitment; Date of Registration)			Protection Efficacy	Storage Requirement during Transport [100]	Dose	Authorization Status (Date)
			Clinical Trial Phase 1	Clinical Trial Phase 2	Clinical Trial Phase 3				
BBIBP-CorV	Inactivated vaccine	Beijing Institute of Biological Products Co., LTD./Sinopharm	ChiCTR2000032459 (China; 29.04.2020)		NCT04560881 (Argentina; 23.09.2020) NCT04795414 (China; 12.03.2021) NCT04510207 (Bahrain, Egypt, Jordan, United Arab Emirates; 12.08.2020)	78.1% [101]	2–8 °C	2	Conditional marketing authorization (30.12.2020)
Corona Vac (PiCoVacc)	Inactivated vaccine	Sinovac Biotech	NCT04352608 (China; 20.04.2020) NCT04383574 (China; 12.05.2020) NCT04551547 (China; 16.09.2020)		NCT04456595 (Brazil; 02.07.2020)	Brazil: symptomatic prevention: 50.4%; mild cases prevention: 78%; Severe cases prevention: 100% Turkey: 83.5% Indonesia: 65.3% (confidence interval not reported) the United Arab Emirates: 86% [102]	Room temperature	2	Conditional marketing authorization (05.02.2021)
New Crown COVID-19	Inactivated vaccine	Wuhan Institute of Biological Products/Sinopharm	ChiCTR2000031809 (China; 11.04.2020)		ChiCTR2000034780 (The Union Arab Emirates; 18.07.2020) ChiCTR2000039000 (Morocco; 13.10.2020) NCT04612972 (Peru; 03.11.2020) NCT04510207 (Bahrain, Egypt, Jordan, United Arab Emirates; 12.08.2020)	72.8% [101]	2–8 °C	2	Conditional marketing authorization (25.02.2021)
Ad5-nCoV	Viral vector vaccine	CanSino Biologics Inc. & Academy of Military Medical Sciences, PLA of China	ChiCTR2000030906 (China; 17.03.2020) NCT04313127 (China; 18.03.2020)	ChiCTR2000031781 (China; 10.04.2020) NCT04341389 (China; 10.04.2020)	ChiCTR2100044249 (Pakistan, Russia, Argentina, Chile, Mexico; 12.03.2021) NCT04526990 (Pakistan, Russia, Argentina, Chile, Mexico; 26.08.2020)	65% for preventing symptoms, 90% effective at preventing severe symptoms [103]	2–8 °C	1	Conditional marketing authorization (25.02.2021)
ZF2001	Recombinant protein subunit vaccine	Anhui Zhifei Longcom Biopharmaceutical	ChiCTR2000035691 (China; 16.08.2020) NCT04445194 (China; 24.07.2020)	NCT04466085 (China; 10.07.2020)	ChiCTR2000040153 (China; 22.11.2020) NCT04646590 (China, Ecuador, Indonesia, Pakistan, Uzbekistan; 30.11.2020)	Not available at the time of writing	2–8 °C	2–3	Emergency use authorization (10.03.2021)
KCONVAC	Inactivated vaccine	Shenzhen Kangtai Biological Products Co., LTD. & Beijing Minhai Biotechnology Co., LTD.	ChiCTR2000038804 (China; 02.10.2020) NCT04758273 (China; 17.02.2021)	ChiCTR2000039462 (China; 28.10.2020) NCT04756323 (China; 16.02.2021)	NCT04852705 (21.04.2021)	Not available at the time of writing	2–8 °C	2	Emergency use authorization (07.05.2021)

## Data Availability

Not applicable.
